# Embolic Agent Selection and Outcomes in Renal Angiomyolipoma Embolisation

**DOI:** 10.1111/1754-9485.70108

**Published:** 2026-04-22

**Authors:** Ross O'Shea, Himanshu Pendse, Kevin Ho, Hasan Hasan, Ross Vander Wal, Matthys Van Wyk, Jonathan Tibballs, Gurjeet Dulku

**Affiliations:** ^1^ Medical Imaging and Interventional Radiology Department Fiona Stanley Hospital, South Metropolitan Health Service Perth WA Australia; ^2^ Radiology Department Royal Perth Hospital Perth WA Australia; ^3^ Radiology Department Sir Charles Gairdner Hospital Perth WA Australia

**Keywords:** angiomyolipoma, embolic agents, interventional radiology, renal artery, therapeutic embolization

## Abstract

**Purpose:**

To evaluate whether embolic agent selection influences outcomes following selective arterial embolisation (SAE) of renal angiomyolipomas (r‐AMLs) and to assess procedural safety and effects on renal function.

**Materials and Methods:**

A retrospective observational study was performed of all r‐AML SAEs undertaken at a tertiary institution between 2015 and 2024. Change in tumour size on pre‐ and 3–6‐month post‐procedural imaging was the primary outcome. Secondary outcomes included technical success, re‐intervention rates, complications (Clavien–Dindo), renal function change and length of hospital stay. Outcomes were compared across embolic agents including microparticles, liquid embolic agents, coils and combined approaches.

**Results:**

Forty‐nine patients (51 lesions) underwent angiography, with SAE attempted in 54 procedures. Technical success was achieved in 91%. Tumour size reduction occurred in 91% of lesions with follow‐up imaging, with a mean reduction of 1.75 cm (*p* < 0.0001). Liquid embolic agents alone demonstrated the greatest mean tumour reduction with a mean reduction of 2.4 cm and a 0% re‐intervention rate. Microparticles alone achieved significant size reduction with a low re‐intervention rate, while coil‐only embolisation was associated with inferior outcomes and higher re‐intervention rates. Combined embolisation strategies achieved significant tumour reduction but did not outperform liquid agents alone. Complications occurred in 10% of patients and were predominantly minor. No patient required renal replacement therapy, and the median eGFR did not change significantly.

**Conclusion:**

SAE is a safe and effective treatment for r‐AML in both elective and emergency settings. Liquid embolic agents achieved the greatest tumour reduction with low re‐intervention rates, while coil‐only embolisation showed inferior outcomes and should be avoided where possible.

**Abbreviations**
AML: Angiomyolipoma
CKD: Chronic kidney disease
CT: Computed tomography
ECOG: Eastern Cooperative Oncology Group
eGFR: Estimated glomerular filtration rate
IQR: Interquartile range
MRI: Magnetic resonance imaging
r‐AML: Renal angiomyolipoma
SAE: Selective arterial embolisation
SD: Standard deviation
STROBE: Strengthening the Reporting of Observational studies in Epidemiology
US: Ultrasound

## 1 Introduction

Renal angiomyolipomas (r‐AMLs) are benign renal neoplasms, of which approximately 80% are sporadic, and 20% occur in association with phakomatoses, most commonly tuberous sclerosis complex [1, 2, 3]. Sporadic r‐AMLs are typically solitary, unilateral and more prevalent in females [4]. Histologically, r‐AMLs are composed of dysmorphic blood vessels, smooth muscle and adipose tissue. Their high fat content allows reliable diagnosis on ultrasound (US), computed tomography (CT) or magnetic resonance imaging (MRI) [2, 3]. Renal cell carcinoma remains an important differential diagnosis, particularly in fat‐poor lesions, and should be excluded when imaging findings are equivocal [3].

Low‐risk r‐AMLs can be managed conservatively with active surveillance. Lesions at higher risk of haemorrhage should be considered for nephron‐sparing intervention, including selective arterial embolisation (SAE) or partial nephrectomy [5, 6]. While tumour size > 4 cm has traditionally been used as a treatment threshold, contemporary evidence supports intervention for smaller lesions when intralesional aneurysms ≥ 5 mm or symptoms are present [5, 7, 8]. Tumour size, vascularity and intralesional aneurysms are recognised predictors of haemorrhage. Intervention aims to prevent complications such as retroperitoneal haemorrhage and loss of renal function due to parenchymal destruction or acute bleeding [5].

SAE is an established treatment for r‐AMLs with hemorrhagic risk or active bleeding. Prior studies have demonstrated effective tumour size reduction with preservation of renal function and low complication rates [9, 10, 11, 12, 13, 14, 15, 16]. Most published series report heterogeneous use of embolic agents, and direct comparisons between agents remain limited [17].

The aim of this study was to evaluate whether embolic agent selection influences outcomes following r‐AML embolisation. The secondary aim was to assess the safety of SAE and its effect on renal function.

## 2 Methods

**FIGURE 1:**
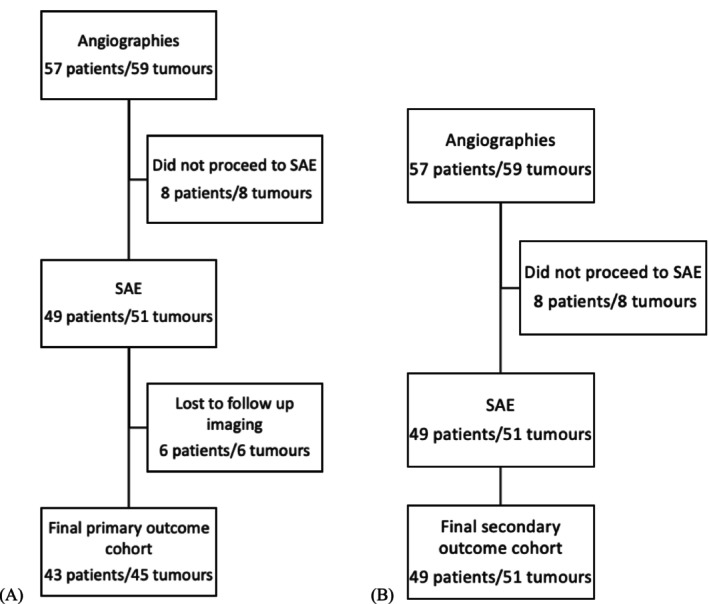
Flow chart of study cohort for primary (A) and secondary (B) outcome measures.

A retrospective observational study of all r‐AML SAEs performed between 2015 and 2024 at a tertiary academic institution was conducted. All patients undergoing SAE were included. Pre‐procedural imaging consisted of US, CT and/or MRI. Patients with available 3–6‐month post‐procedural imaging were included in the primary outcome cohort. Tumour size was measured on pre‐ and post‐procedural imaging by experienced radiologists. All patients undergoing SAE had pre‐procedure blood tests including estimated glomerular filtration rate (eGFR). Post‐procedure blood tests were analysed for changes in eGFR. In patients with changes in eGFR, all available follow‐up blood tests were analysed to assess for resolution over time. Primary and secondary outcome cohorts are outlined in Figure 1.

Performance status was assessed using the Eastern Cooperative Oncology Group (ECOG) scale [18]. Complications were classified according to the Clavien–Dindo system [19]. Length of hospital stay was recorded from the digital medical record.

This study was carried out in accordance with the Strengthening the Reporting of Observational studies in Epidemiology (STROBE) checklist [19].

### 2.1 Statistical Analysis

Categorical variables were summarised using counts and percentages. Continuous variables were expressed as means ± standard deviation (SD) or medians with interquartile ranges (IQR), depending on distribution. Changes in tumour size were analysed using paired Student's *t*‐tests, and changes in eGFR were assessed using the Wilcoxon signed‐rank test. Statistical significance was defined as *p* < 0.05.

### 2.2 Ethical Considerations

Ethical approval with intent to publish was granted by the Quality Improvement Imaging Services Committee at Fiona Stanley Hospital Group with approval number 58,003. Written informed consent for the procedure was obtained from all patients prior to SAE.

## 3 Results

Fifty‐seven angiographies for r‐AML were performed during the study period. Forty‐nine patients (mean age 52.9 ± 16.5 years) were included in the study. Eighty‐six percent (*n* = 42) of patients were female. Ten percent (*n* = 5) of patients had a current diagnosis of tuberous sclerosis at the time of embolisation. ECOG performance status was 0 in 90% of patients, 1 in 8% and 3 in 2%. At the time of SAE, 34 patients had one r‐AML lesion, 5 had two, 3 had three, 1 had four and 6 had more than five lesions. Biopsy of fat‐poor lesions with concerns for renal cell carcinoma was performed in 4% (*n* = 2) of patients, which confirmed the diagnosis of r‐AML in both cases. Patient demographics are summarised in Table 1.

**TABLE 1:** Demographic data.

Demographic data (*n* = 49)	Total (n)	Percentage (%)
Gender		
Male	7	14
Female	42	86
Age bracket		
10–19 years	1	2
20–29 years	2	4
30–39 years	8	16
40–49 years	9	18
50–59 years	13	27
60–69 years	9	18
70–79 years	5	10
80–89 years	1	2
90–99 years	1	2
Tuberous sclerosis		
Yes	5	10
No	44	90

A total of 51 r‐AML lesions with a range of 3–18 cm and a median (IQR) max diameter of 6 cm (3 cm) were treated across 49 patients. Four percent (*n* = 2) had two lesions treated at the time of SAE. Twenty‐four percent (*n* = 12) underwent emergency SAE. All emergency cases presented with pain and signs of retroperitoneal haemorrhage and/or haematoma formation on pre‐procedure imaging. R‐AML lesions that underwent an emergency SAE had a range of 3–14 cm and a median (IQR) max diameter of 6.5 cm (3.5 cm). Seventy‐six percent (*n* = 37) underwent an elective SAE. All 37 elective patients were asymptomatic at the time of SAE. R‐AML lesions that underwent an elective SAE had a range of 3–18 cm and a median (IQR) max diameter of 5 cm (2 cm).

Microparticle SAE alone represented 63.27% (*n* = 31). Microparticles comprised trisacryl gelatin, cross‐linked polyvinyl alcohol, sulfonated acrylamide‐modified polyvinyl alcohol or polymethylmethacrylate with a polyzene‐F surface coating. Microparticle size ranged from 100 to 500 μm. Lesions that underwent SAE with microparticles alone had a lesion range of 3–18 cm and a median (IQR) max diameter of 5 cm (2 cm). Mean change in max diameter (± SD) pre and post SAE was 1.6 cm (±1 cm) with a *p*‐value of 0.0001.

Liquid embolic agents (ethanol–lipiodol emulsion) alone represented 12.24% (*n* = 6). Ethanol–lipiodol emulsion was prepared at ratios of 5:1 or 7:3. Lesions that underwent SAE with liquid embolic agents alone had a lesion range of 4–7 cm and a median (IQR) max diameter of 4.5 cm (2 cm). Mean change in max diameter (± SD) pre and post SAE was 2.4 cm (±1.1 cm) with a i‐value of 0.0083.

Coils alone represented 6.12% (*n* = 3). They were used in the emergency setting (*n* = 2) due to acute rupture and once in the elective setting due to the need to avoid an arteriovenous fistulous component to the AML lesion. Lesions that underwent SAE with coils alone had a lesion range of 5–14 cm and a median (IQR) max diameter of 6 cm (9 cm). Mean change in max diameter (± SD) pre and post SAE was 2.2 cm (±3.2 cm) with a *p*‐value of 0.3505.

Microparticle and liquid embolic agents used concurrently represent 6.12% (*n* = 3). Ethanol–lipiodol emulsion was used as the liquid agent twice, and glue was used once. These lesions had a range of 6–12 cm and a median (IQR) max diameter of 8 cm (6 cm). Mean change in max diameter (± SD) pre and post SAE was 1.5 cm (±0.7 cm) with a *p*‐value of 0.0219.

Microparticles and coils utilised together represented 12.24% (*n* = 6). These lesions had a range of 4–8 cm and a median (IQR) max diameter of 7.5 cm (2 cm). Mean change in max diameter (± SD) pre and post SAE was 2 cm (±0.7 cm) with a *p*‐value of 0.0013. Baseline lesion characteristics and embolic agent selection are summarised in Table 2. The mean change in tumour size according to the embolic agent is summarised in Table 3.

**TABLE 2:** Median max diameter pre‐SAE and embolisation agent used.

Embolisation agent	Percentage of SAE (*n* = 49)	Median (IQR) max diameter
Particles	63.27% (*n* = 31)	5 cm (2 cm)
Liquid (Ethanol–lipiodol emulsion)	12.24% (*n* = 6)	4.5 cm (2 cm)
Coils	6.12% (*n* = 3)	6 cm (9 cm)
Particles and liquid Ethanol–lipiodol emulsion (*n* = 2) Glue (*n* = 1)	6.12% (*n* = 3)	8 cm (6 cm)
Particles and coils	12.24% (*n* = 6)	7.5 cm (2 cm)

**TABLE 3:** Mean change in max diameter post SAE and embolisation agent used.

Embolisation agent	Mean (± SD) Change post SAE	p
Particles	1.6 cm (±1 cm)	0.0001
Liquid (Ethanol–lipiodol emulsion)	2.4 cm (±1.1 cm)	0.0083
Coils	2.2 cm (±3.2 cm)	0.3505
Particles and liquid Ethanol–lipiodol emulsion (*n* = 2) Glue (*n* = 1)	1.5 cm (±0.7 cm)	0.0219
Particles and coils	2 cm (±0.7 cm)	0.0013

Twelve percent (*n* = 6) were lost to follow‐up. Ninety‐one percent (*n* = 41) in which follow‐up scans were available showed reductions in tumour size. Four and a half percent (*n* = 2) showed increased tumour size at 3–6‐month follow‐up, and an additional 4.5% (*n* = 2) showed no change in tumour size. Post SAE r‐AML lesions had a range of 1.2–16 cm and a median (IQR) max diameter of 4.3 cm (2.5 cm). Tumour size post SAE decreased by a mean of 1.75 cm [95% CI 1.39–2.12] with a *p*‐value of < 0.0001.

Technical success, defined as successful selective catheterisation of the tumour feeding artery with delivery of the embolic agent, was achieved in 91% (*n* = 49) of cases. Five SAEs were abandoned due to vasospasm or inability to access sub‐segmental arteries. Three of these patients underwent successful re‐embolisation, while two required no further intervention. Three angiographies did not proceed to SAE as there was no tumour vascularity at the time of SAE.

**FIGURE 2:**
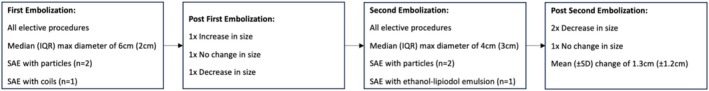
Re‐intervention characteristics.

Seven percent (*n* = 3) of lesions underwent subsequent treatment post SAE. The characteristics of these lesions are outlined in Figure 2. The size of these lesions ranged from 4 to 6 cm with a median (IQR) max diameter of 6 cm (2 cm). Two of these patients underwent SAE with microparticles originally, and one patient underwent SAE with coils. Subsequent SAE was performed with microparticles in two of these patients, and one patient underwent subsequent SAE with a liquid embolic agent. Liquid embolic agents used alone had a re‐intervention rate of 0%. Microparticle alone had a re‐intervention rate of 6.45%, and coils used alone had a re‐intervention rate of 33%. Embolisation agents used concurrently all had a re‐intervention rate of 0%.

**FIGURE 3:**
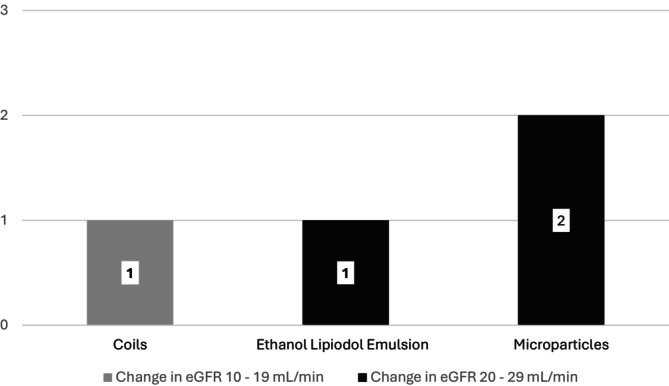
Number of patients with changes in eGFR post SAE.

Ten percent (*n* = 5) of patients experienced post SAE complications. Four of these complications were classified as Clavien–Dindo grade I and one was classified as grade II [20]. All grade I complications represented reductions in eGFR > 10 mL/min/1.73 m^2^ without need for intervention. These lesions had a range of 5–14 cm with a median (IQR) of 6 cm (4.5 cm). Reductions in eGFR occurred post embolisation with microparticles (300–500 μm used alone and concurrent 100–300 μm with 300–500 μm) alone, liquid embolic agents (ethanol–lipiodol emulsion) alone and coils alone and represented 50%, 25%, and 25% of grade I complications, respectively. Reductions in eGFR to a higher CKD stage occurred in three of the four patients. These reductions did not improve but were stable on long‐term data. Median eGFR did not differ significantly pre‐ and post‐procedure (*p* = 0.424), and no patients required renal replacement therapy. The distribution of post‐procedural renal function changes is shown in Figure 3. The grade II complication lesion was 8 cm and occurred post SAE with microparticles and coils concurrently. This patient suffered a haemorrhage from embolisation site seven days post‐procedure. This complication was treated conservatively without the need for further embolisation or surgical intervention.

Length of hospital stay post elective SAE ranged from 0 to 1 day with a median (IQR) of 0 (1). Length of hospital stay post emergency SAE ranged from 0 to 15 days with a median (IQR) of 2.5 (2).

## 4 Discussion

The primary objective of this study was to evaluate whether embolic agent selection affects outcomes in r‐AML embolisation. Microparticles (100–300 μm and 300–500 μm Embospheres) were the most frequently used embolic material and resulted in a significant reduction in tumour size. Liquid embolic agents (ethanol–lipiodol emulsion) demonstrated a greater mean tumour size reduction, suggesting particular efficacy in selected anatomical and clinical contexts. This finding is consistent with previous studies evaluating liquid embolic agents [21, 22] and with meta‐analyses showing superior tumour shrinkage and lower re‐intervention rates compared with other embolisation agents [23].

The frequent use of microparticles in this cohort is notable, as traditional teaching has favoured liquid embolic agents such as ethanol–lipiodol, particularly in lesions with arteriovenous shunting where distal penetration is desired. This observation likely reflects real‐world variation in operator preference and case selection, including differences in vascular architecture and procedural objectives.

Coils used alone did not achieve a statistically significant reduction in tumour size. This may relate to the more proximal nature of coil embolisation, increasing the likelihood of collateral reperfusion, as well as the small number of coil‐only procedures. Notably, coil‐only embolisation was not performed during the latter half of the study period. Multimodal embolisation approaches combining embolic agents demonstrated promising results. Both microparticles combined with liquid embolic agents and microparticles combined with coils resulted in significant tumour size reduction. Although patient numbers were small, these findings suggest combined approaches may enhance embolisation efficacy by targeting multiple vascular components of the tumour. Previous studies have reported no statistically significant difference in tumour diameter reduction between embolic agents [24], highlighting the need for larger comparative studies to define optimal agent selection across clinical scenarios.

The technical success rate of 91% is consistent with prior reports [10, 11, 12, 21, 23]. Factors limiting technical success included vasospasm, complex vascular anatomy and difficulty accessing small segmental arteries for super‐selective embolisation.

A key secondary objective was assessment of SAE safety. Post‐procedural complications were infrequent and predominantly mild (Clavien–Dindo grade I) [20]. A reduction in eGFR > 10 mL/min/1.73 m^2^ occurred in a small cohort, with some patients progressing to a higher CKD stage. These reductions did not recover but remained stable on long‐term follow‐up. Renal replacement therapy was not required in any patient. Renal impairment occurred across different embolisation agents and was not statistically significant. One grade II complication (delayed post‐embolisation haemorrhage) was managed conservatively without surgical intervention. Overall, these findings align with previous studies demonstrating SAE to be a safe nephron‐sparing intervention with no significant long‐term impact on renal function [9, 10, 11, 21].

Liquid embolic agents and concurrent embolization strategies were effective in reducing tumour size while maintaining low re‐intervention rates, consistent with meta‐analytic data [23]. In contrast, coils demonstrated the lowest tumour reduction and highest re‐intervention rates. Prior studies have cautioned against coil‐only embolisation due to increased distal collateral formation and the potential to complicate future re‐embolisation, and their use should therefore be limited where possible [25, 26].

A notable distinction in this cohort was between emergency and elective SAE. Emergency cases uniformly presented with symptomatic retroperitoneal haemorrhage or haematoma and had larger tumours requiring urgent haemorrhage control. Elective cases involved asymptomatic but high‐risk lesions, with treatment decisions guided by tumour size (> 4 cm), intralesional aneurysms (≥ 5 mm) and rupture risk [6, 7, 8]. Embolic agent selection differed between groups, with coils or coils combined with microparticles used more commonly in emergency settings, and microparticles alone predominating in elective cases. Coil use in the elective setting was uncommon. Both emergency and elective groups demonstrated significant tumour size reduction post‐SAE. Emergency cases, however, required longer hospital stays, largely reflecting clinical severity and non‐procedural factors such as disposition planning. Complication rates were comparable between groups, and outcomes were favourable overall. Re‐intervention rates were low, particularly in emergency cases, though the relatively short follow‐up period may account for lower rates compared with prior reports [9, 15]. No significant differences were observed in tumour reduction, complications or re‐intervention rates, consistent with previous comparisons of elective and emergency SAE [22, 27].

Overall, these findings reinforce the established efficacy of SAE for r‐AML. Variability in embolic material selection and technique underscores the need for further comparative research. In the absence of clear embolic superiority, cost considerations are increasingly relevant, and formal cost‐effectiveness analyses specific to r‐AML embolisation are warranted [28, 29, 30, 31]. Longer‐term follow‐up beyond 12 months would further clarify the durability of tumour control and renal function preservation.

Limitations of this study include its retrospective observational design, which introduces potential selection bias and limits causal inference. Treatment allocation was also likely influenced by operator preference and lesion characteristics. The modest sample size, loss to follow‐up in 12% of patients and relatively imbalanced group sizes further limit the robustness of comparisons. In addition, there was substantial heterogeneity in embolisation technique and embolic agent selection, which may have influenced outcomes. Unmeasured confounders, including tumour vascular architecture and operator‐dependent factors, may also have contributed to the observed differences. Accordingly, these findings should be interpreted as observational associations rather than definitive evidence of superiority. Prospective, standardised comparative studies and cost‐effectiveness analyses are warranted.

## 5 Conclusion

SAE is a safe and effective treatment for r‐AML in both elective and emergency settings. Liquid embolic agents achieved the greatest tumour size reduction with low re‐intervention rates, while coils alone were associated with inferior outcomes and should be avoided when possible. Embolic agents used concurrently were not shown to be more efficacious than using liquid embolic agents alone. SAE for r‐AMLs is a safe intervention with a low number of severe complications and no significant effect on renal function.

## Author Contributions

**Matthys Van Wyk:** writing – review and editing, resources. **Ross Vander Wal:** writing – review and editing, resources. **Gurjeet Dulku:** conceptualization, writing – review and editing, supervision, methodology, validation, resources. **Ross O'Shea:** investigation, writing – original draft, visualization, writing – review and editing, formal analysis, data curation, methodology, project administration, software. **Himanshu Pendse:** writing – review and editing, supervision, resources. **Jonathan Tibballs:** writing – review and editing, resources. **Kevin Ho:** writing – review and editing, resources. **Hasan Hasan:** writing – review and editing, resources.

## Funding

The authors received no specific funding for this study.

## Ethics Statement

Ethical approval with intent to publish was granted by the Quality Improvement Imaging Services Committee at the Fiona Stanley Hospital Group (approval number 58003). Written informed consent for the procedure was obtained from all patients prior to selective arterial embolisation.

## Consent

The authors have nothing to report.

## Conflicts of Interest

The authors declare no conflicts of interest.

## Data Availability

The data that support the findings of this study are available from the corresponding author upon reasonable request.
